# British Yemeni Young People’s Experience of Body Image, Home, Food, Language, and Religion

**DOI:** 10.1007/s43151-022-00089-1

**Published:** 2023-03-13

**Authors:** Huda Kamel Ahmed

**Affiliations:** School of Environment, Education, and Development, Manchester Institute of Education, Manchester, UK

**Keywords:** British Yemeni, Conduct of everyday life, Intersectionality, Cultural hybridity, Post-colonialism

## Abstract

This paper provides an in-depth knowledge on the social practices of an under-researched ethnic group in the UK, the British Yemenis. Through an exploration of their lived activities in the context of the conduct of everyday life, this study uses photo-novella and semi-structured interviews in combination, to present five main themes which emerged from the study. These are body image, food, home, language, and religion. The paper shows how British Yemeni young people connect and disconnect, at different levels, times, and context, with mainstream British cultures and Yemeni subcultures, displaying elements of cultural hybridity that are unique to these individuals, yet at the same time provides some information on the Yemeni community. The study follows the daily activities of six British Yemeni young people in a longitudinal manner, exploring an understanding of how social structures and cultures have, and are continuously, impacting the young people’s conduct of everyday life. The study also contributes to the use of intersectionality and post-colonialism as analytic tools in the study of young people’s lived experiences.

## Introduction

Yemenis are an under-represented ethnic group in Britain despite being the earliest Muslim settlers in Britain since the nineteenth century (Sanni 2014). There have been numerous studies on the diaspora of other ethnic minority groups in Britain such as the Pakistanis (Zriba 2018), Indians (Ghai and Desai 1964), and Africans (Killingray 1993). The situation of other immigrants, who perhaps may be in similar cultural, social, political, and economic situations as the Yemenis, may also be researched in other cultural and historical studies around diaspora and migration, and people of different cultures may have similar cross-cultural applicability (Glaser 1958; Agishtein and Brumbaugh 2013). However, the focus in this article is on the diaspora related to British Yemenis.

The Yemeni diaspora developed as a result of different historical waves of migration (Sanni 2014; Guns 2010). The current war in Yemen (Kaptan 2021; Hokayem and Roberts 2016) has increased Yemeni diaspora mobilisation, and this has facilitated their visibility and voice as immigrants globally (Moss 2021). Some Yemenis migrated to the UK during British colonisation (Roochnik 2001), while others migrated to better their economic or educational status (Civantos 2015). However, due to the ethnic, religious, and linguistic diversity within the Arab world (of which Yemen is part) and those settling in Britain, information on the numerical strength and spatial concentrations of Arab communities is hampered by the lack of accurate quantitative data (El-Solh 1992). The “Arab” ethnicity category was added to the 2011 UK Census, and figures estimate that there are approximately 70–80,000 people of Yemeni origin who were living in Britain (White 2011). One of the reasons behind the lack of a precise number is due to the ethnic grouping in these censuses. The recent 2021 census has an option for ethnic categorisation in which a person can note their ethnicity using their own words (ONS 2022), and so there is hope that this census will provide a clearer picture when its results are released. Literature on the Yemeni community in Britain is also limited. There is some work on the religious and social solidarity of the Yemeni community in Britain (Dahya 1965) as well as the attempts of early Yemeni settlers to integrate into British society (El-Solh 1992; Sabagh 1994; Sanni 2014; Stevenson 1993).

This paper provides knowledge on the experiences of current British Yemeni young people in Britain. Through examining their actions and activities in an in-depth way, the study explores the connections in the structures, cultures, and relations of the young people, suggesting a notional link to their personality (Dreier 2011) and personhood (Roth 2016) in the context of the conduct of everyday life. The study gives examples of how young people (of first, second, and third generations) live their daily lives within the social and cultural framework of British society, while at the same time (albeit at different levels) preserving their Yemeni histories and practices. In this article, I argue that it is in the study of their conduct of everyday life that researchers can understand such participations and interactions of structures and cultures.

### Conceptual Framework

#### The Conduct of Everyday Life

The conduct of everyday life articulates the subjective experiences involved in people’s creation and organisation of their activities and tasks, as well as the way in which they participate in and across different social contexts (Holzkamp 2015) in the fabric of everyday life (Dreier 2007). The concept roots its inquiry in the meanings and reasons for action, contributing to the dialogues among people on how to live in a contemporary society (Kristensen and Schraube 2014). Here, I focus on the daily activities which six British Yemeni young people engage in and which I take to represent their personality (Dreier 2011). Instead of focusing on a simple explanation of the causal impact society and the social arrangements it has on young people’s activity, I explore the interweaving of relational activity that both contributes to and emerges out of the culture and structures of certain institutions. This leads to an evolving sense of continuity and change for the young person. I take the position that a person is both a subject and subjected to the society in which he/she lives in and that agency and activity are demonstrated through the person’s living and being. This suggests a hybridity in a young person’s evolving personhood that is conterminously the same and different to others associated with the Yemeni ethnic group.

#### Post-Colonial Concerns and Hybridity

Hybridity is an umbrella term for different forms of blending, mixing, and combining (Mäntynen and Shore 2014; Pieterse 2001), and the focus in this study is as it relates to the blending of different social practices that perhaps contain elements of ethnic and national cultures which the person participates in and engages with. This is of great importance when studying people that are possibly affected by post-colonisation, and the movement out of their ancestral homeland to a host country, as is the case with British Yemenis. This study makes connections to post-colonial theory in order to understand some of the factors which have perhaps contributed to the formation of the cultural hybridity of the young people. Furthermore, the world is more connected than ever before, and so the hybridity in a person’s evolving personhood may be influenced by the increase in ethnic intergroup contact and migration processes (KagitçibaşLi 1997) of the young people and the increase in new technology in contemporary globalisation that may enable new phases of intercultural contact (Hrynyshyn 2002). As a result of this, several interdisciplinary fields have extensively researched explanatory concepts, analytical tools, and heuristic devices that help explain the interconnection of social structures and cultures in the formation of cultural hybridity (Schwartz 2005; Sabatier 2008; Desmet et al. 2017; Bhugra and Becker 2005).

#### Intersectionality Lens

Intersectionality, for example, is one way that could be used to explain such interconnections. Often used to theorise identity (Gopaldas 2012), intersectionality studies the relationships among multiple dimensions and modalities of social relationships and subject formations (McCall 2008), including social practices, institutional arrangements, and cultural ideologies and the outcomes of these interactions in terms of power (Davis 2008). It can be used, therefore, to study the interactivity of social identity structures (of race, gender, sexuality, religion, ethnicity, and nationality) in fostering life experiences. The blend of these identity structures also constitutes the hybridity of the young person. In the context of this study, I use intersectionality as an analytical tool. This is because I take the position that people are active subjects, that do, and in their doing, forms of their agency reflect their evolving social structures and cultures. These cultures encompass their tradition, religion, manners, behaviours, and customs (Shah 2016; Zimmermann 2012). All these, and more, are embedded in the daily activities of the young person.

### Research Context and Focus

To appreciate the fullness of our intersecting lives (Baumeister and Muraven 1996), the structures and cultures of everyday living that expose both the social arrangements of Yemeni life in the UK, as part of shifting cultural tropes of British society, must be taken in connection to each other. This study, therefore, contributes to debates around intersectionality by steering them in a direction that centres on the exploration of the daily experiences of British Yemeni young people through focusing on the act of doing. In this way, the relational and interconnected experiences of these young people will be presented.

By exploring the daily experiences of six British Yemeni young people in Birmingham, UK, through their interconnected activities, I move away from traditional identity theories or personality paradigms as they appear to provide an essentialised and perhaps a static notion of who the young people are, and are becoming, that may not fully engage with the evolving nature of their daily living. Instead, I explore a more complex relational, pragmatic, interconnected, historical, cultural set of social arrangements and practices. In this way, I provide a portrait of the social construction of British Yemeni young people’s hybrid and evolving cultures and structures. I do not speculate that all British Yemeni young people are alike, but by understanding some of the nuance around such community, their history-culture-of activity which they are part and reproduce as Yemeni society can be identified. While such experience is not unique to British Yemenis, the way in which they interconnect their living experiences of culture and structure provides a contribution to the body of sociological research that focuses on issues of ethnicity and diaspora.

## Methods and Material

The data, presented in this article, is drawn from a larger doctoral study of six British Yemeni young people, conducted over nineteen months, documenting the types of everyday experiences of these young people as well as the different forms of learning and development they experience over time. In using longitudinal studies, I was able to capture the dynamic and idiosyncratic nature of their lived experiences (Caruana et al. 2015) and reflect on some of the complex and personal stories of such experiences, identifying related events to particular exposures, with regards to presence, timing and chronicity (Ruspini 2002; Cuervo and Cook 2020).

### Participants

The participants were recruited through gatekeepers and through my own engagement—as a British Yemeni myself—in the Yemeni community in Birmingham. The participation criteria included that the young people were (1) between the ages of 16–19 and so mature enough to reflect on experiences, (2) attended formal education in England to ensure some aspects of their homogeneity, (3) currently living in Birmingham to enable face to face research, and (4) available to contact over the 2-year period to complete date collection over the period of research. Six young people took part in the study, and although this number was small, it allowed an in-depth account of each participant daily lived experiences. The participants included 3 young women, *Fatima*, *Nuha*, and *Sausan*, and 3 young men, *Younus*, *Rashad*, and *Adam* (all names are pseudonyms). All participants indicated during the interviews that they are ethnically Yemeni and nationally British. *Fatima* and *Nuha* are first-generation settlers, with their parents moving to Britain in their early 20 s and settling for work purposes. *Younus* and *Rashad* are second-generation, their parents born in the UK. *Sausan* and *Adam* are third-generation settlers, with their grandparents arriving due to colonisation. All participants identified themselves as being Muslim.

### Methods

Photo-novella (Wang and Burris 1994) and semi-structured interviews (Newton 2010) were used in combination to capture the young people’s experiences. I gave each participant one disposable camera and asked them to take photos of their daily activities on 4 non-consecutive days. Two of the days were routine days, to show the familiar and intimate sedimentations of their preferred way of conducting of life (Dreier 2011), and 2 days were non-routine, perhaps a weekend, to provide a more particular variation on the regular conduct of everyday life (Hybholt 2015). This provided an assessment of contiguous episodes over each full day.

The photos taken by the participants were used as a stimulus to narrate their daily experiences (Hurworth 2003). Giving the control of the camera to the young people not only empowered them, but also offered them the chance to amplify their place in and experiences of the world, and so they were able to narrate their personal experiences and conditions as they see them (Wang and Burris 1994). This provided an interpretation, purpose, and/or message from the participants’ own unique perspective and experience (Wang and Burris 1994). Using photography also generated dialogue in a way that captured rich conversations and deep discussions, longer elicitation, and more comprehensive interviews (Freire 2013; Collier 1957). Semi-structured interviews were then followed so that participants could elaborate on the photographs taken (Cohen and Crabtree 2006). Photo-novella and semi-structured interviews, used in combination, created a visual expression of the social and cultural constructs of the participant’s agency and structure (Booth and Booth 2003), allowing the voices and visions of key elements in the young people’s everyday lives to be revealed.

This process was then repeated 12–19 months later, and the period between the first and second set of interviews depended on the availability of the participants and the possibility of meeting considering COVID-19 restrictions. Between March 2019 and October 2020, a total of 24 semi-structured interviews were conducted, each participant taking part in four in-depth interviews of approximately 45–60 min each. The study was conducted in accordance with the Code of Practice of the University of Manchester and in accordance with the guidelines of the British Education Research Association (Hammersley and Traianou 2012). All participants signed a consent form.

### Analysis

The semi-structured interviews were transcribed and analysed thematically, capturing patterns within the data (Braun et al. 2019; Nowell et al. 2017). Specifically, a hybrid approach of qualitative methods of thematic analysis was used following guidance from Fereday and Muir-Cochrane (2006) incorporating a deductive, priori template of codes approach outlined by Crabtree and Miller (1999) and the data-driven inductive approach of Boyatzis (1998).

The first part of this hybrid approach is a deductive content directed approach in which I used Dreier’s theory of a person (Dreier 2011) to guide and form the codes for analysis (Hsieh and Shannon 2005; Assarroudi et al. 2018; Crabtree and Miller 1999). This theory is presented through a study of person-situation-activity approach, with reference to:

1. The *order and arrangement*—these include the social context (place), social practices (activities—concerns, demands and obligations, purpose and meaning, family history, and practices—and relation), and social arrangements (order and time noticing rhythms and shifts) of the participant.
2. The *situated participation and movement*—these include the sequence of events (which builds the idea of the habits a person has and develops) and perspective of experiences (which narrate possible changes and reflections in the person’s activities).
3. The *conduct of everyday life*—which both (1) and (2) contribute to, providing a portrait of the young person’s evolving biography.

These codes were generated directly from Dreier’s paper: “Personality and the conduct of everyday life” (Dreier 2011).

The second hybrid approach is a data-driven inductive approach, where emerging themes become the categories for further analysis (Boyatzis 1998). Considering the codes offered by the deductive approach (using Dreier’s theory), I then re-read and examined each of the narratives. As this paper focuses on the social practices that were similar for all six participants, I particularly concentrated on practices that were predominantly influenced by the participants’ family’s histories and practices. I then identified patterns that emerged from the six narratives. This offered a way of understanding the interconnections between the structures and cultures shared by my six participants. To reduce the complexity of data, and facilitate coding for all 24 transcripts, I used NVivo software technology (AlYahmady and Al Abri 2013). This is as an analytical tool, designed for qualitative data analysis to manage the coding procedure for the subjectivity, richness, and comprehensive text-based information of the data (Trigueros Cervantes et al. 2016).

The relationships between categories and themes of data led to the main points of discussion for the analysis. To document the similar experiences of the participants, themes emerge directly from the narratives. Connecting Dreier’s codes for the deductive approach with the thematic codes for the inductive approach provided vital information on the similarities and differences between the six participants. This provides a justifiable understanding of some of the heterogeneity, as well as some of the homogeneity, of everyday life experiences of British Yemeni young people. For the purpose of this paper, the data analysed concentrates on the connections across British Yemeni young people’s lives, and not the individual stories of the young people, and so thematic analysis was effective in answering questions on such experiences. Bringing together components of the participants’ experiences provides a flexible and more interpretative and conceptual orientation to the data (Nowell et al. 2017), connecting social context, social practices, and social arrangements to the cultures, structures, and relations of the participant individually and the group of young people, as a whole.

## Findings

Five main themes—body image, food, home, language, and religion—emerged from the data. Although these are general categorising themes, there are subtle differences within each theme showing the uniqueness and individuality of the experiences of each participant. I now explore each of these themes, using participants’ references as the heading for each theme.

### Body Image: “I Care About How I Look”

The focus on body image is not a new phenomenon. With the increase in the use of social media, and particularly with reference to pictures of movie stars and fashion models (El Ansari et al. 2010; Goodyear 2020), it is not surprising that the young people in this study also carry out practices related to their body image. Perceptions of beauty for the young people are evaluated by the contemporary society in which they live, which influences their opinions of what is deemed to be fashionable, attractive, and ideal. All six participants reported that a large part of their daily activities consist of taking care of their appearance. Although there are differences in priorities, effort, and influence between the participants, they all showed that they cared about how they looked.

The influence of social media on the perception of body image is portrayed clearly in *Sausan* and *Nuha*’s narratives.

*Sausan: I follow Instagram bloggers and YouTubers. Mainly hijabi ones cos I can relate to them more. They speak to me in a certain way. If I go out in evenings with family, I don’t wear much makeup but if it’s with friends, I do… we all wear makeup. I don’t have friends that don’t wear makeup. It’s the norm. And I also don’t like my skin. I have acne So, yeah, the makeup covers not all my acne but mainly my scars... Sometimes I feel people will probably look at me differently, perhaps. I don’t think I would wear makeup as much if I didn’t have it. It’s pressure from society to look a certain way, I guess.*

*Nuha: I end my day by … washing my face, doing a bit of skin care. Face wash, moisturize. It’s like make myself look less ugly… I have like a disproportionate face but at least I don’t have spots... Dry skin everywhere… which isn’t nice… and I see others on my phone with clear moist skin… So, I moisturize so I don’t look as bad.*

*Sausan* has acne, and so her daily activities involve putting makeup on to cover her facial flaws. She also states that “it is the norm”—all her friends apply makeup, and so this is a social practice. Beauty perception is not only influenced by social media, but also by her social relations, and states that there is “pressure from society” to look a certain way. Sausan follows bloggers and YouTubers that wear the headscarf, because she “relates to” them, and this shows how she integrates elements of her religious obligation to wear the headscarf with her mainstream culture of looking “a certain way”. Nuha, on the other hand, seems dissatisfied with the way she looks and so to make herself “look less ugly”, applying beauty products to avoid having dry skin, or spots, with the hope of having “clear moist skin” like others she sees on her phone.

There is also some mention in *Fatima*’s narrative of looking “better overall”.

*Fatima: I get out of bed around 7, and then I like to get ready, put my makeup on, cos like most girls wear it now and it looks better, …, after I get ready, it usually doesn’t take long as I am getting better at my makeup, then I go down”*

In previous years, prior to attending university, *Fatima* did not wear makeup except during family parties and stated that she was “not so good at putting it on”. Being at university seems to have encouraged her to wear it more frequently, and she is “getting better at” applying it. Fatima shares similar feelings to Sausan, saying “most girls wear it”, and so it is a cultural norm. She also states that “it looks better overall”. A complex range of factors influence body image perception, and this includes socio-demographic factors (gender, age, country) and psycho-social factors (stress, social support, and quality of life) (Burnette et al. 2017). Satisfaction with and concerns about body image are also affected by social norms and cultural standards (Izydorczyk et al. 2020), and this seems to be the case with *Sausan*, *Nuha*, and *Fatima*.

Although dissatisfaction with body shape, image, or looks may be associated more with women than men (Demarest and Allen 2000; El Ansari et al. 2010), it is not unique to them. *Rashad*, *Younus*, and *Adam* are also conscious of their physical appearance, and elements of their daily life reflect this.

*Rashad: I used to be, like, big before…like fat big. Like, last summer holidays I went on a diet. I needed to lose weight, I was, like, obese. I was overeating… I used to have a big appetite. I didn’t like the way I looked. Sometimes people used to make fun of me.*

*Younus: It is organic coconut oil… don’t like chemicals in my hair. You see, it’s easy for my hair to get frizzy and messy, and so I do this to my hair every day to keep the curls looking good, I put it on, and then wash it out. It’s not to the level that I feel insecure, but I like to keep my hair tidy.*

*Adam: I put my headphones on.. It’s just there as an image… I spend a lot of my clothes, cus my taste is different from others, and I like expensive clothes, that fit my image*…

*Rashad* focuses his energy and activities on losing weight, *Younus* ensures his hair is looked after daily, and *Adam* has a particular way in which he wants to display himself, “an image” by having headphones on even though they are switched off. At another time in his narrative, *Adam* also mentions that he spends a large amount of his money on buying specific “clothes that fit” his image. At times, taking care of body image originates from social norms, at other times because of the opinion of others, and at other times because of the young person’s own understanding and perceptions of themselves. *Younus’* self-awareness of what looks good as a hair style, and what does not, is reinforced in his daily activities of applying specific products to his hair. Rashad does not “like the way” he previously looked when he was overweight, and perhaps there is a perception that being fat is frowned upon by the society he is in, and this is reinforced by his statement that at times “people used to make fun” of him. These activities and associated feelings are by no way unique to these individuals and may be an expression of their young age and their subjected to the mainstream society in which they live and participate.

### Food: “Traditional Food Is Now a Bit of Everything”

All participants took photographs of food and discussed it as part of their social practices, with Yemeni cuisine being a main part of their eating habits. Traditional and local foods are a symbol of subjective belongings, customs and traditions, and the relationships that exist in communities (Roudsari et al. 2019; Wahlqvist & Lee 2007; Jordana 2000). *Younus* talked about the cultural practices around food in his family.

*Younus: My mum makes traditional food…The Yemeni way. I usually set the table at home for us to eat together… we eat traditional food usually, like fish and chips and sometimes Yemeni rice and chicken.*

HA: Fish and chips is traditional food?

*Younus: Yeah, its British food and we like to eat it. Traditional is now a bit of everything. We sometimes sit on the floor to eat together as we sometimes eat from one plate, using our hands, and sometimes we eat on the table. It all depends on the day. It depends on the food my mum makes. I like sitting on the floor because it goes back to the feeling of a more natural way, and it is the Sunnah [tradition] of the Prophet.*

*Younus* uses the term “traditional” to show the hybridity of his food culture, where Yemeni food meets English food. *Younus* speaks about sitting on the floor to eat, “the Yemeni way”, and points out that some “cultural Yemeni” cuisines cannot be found on the Internet because “it’s like from the village” and that he must ask his mother for the recipes as they are “passed through [the] generations”. *Younus* also points out some of the ethnic and religious practices around food and the customs around eating. Such practices include eating with the hands, eating from a shared plate, and eating together, all of which are traditional ethnic practices and part of the customary method in Yemen. As *Younus* notes, such practices are associated with Islam and emulate the actions of the Prophet Muhammad (Swarup 2003).

All six participants narrate positive feelings towards Yemeni food, which was usually served at dinner time when family members often got together to socialise after completing their personal daily routines. The food was often ready when the young person returned home, and the type of food cooked varied depending on whether the day was routine or not, whether there is an occasion such as birthday or Eid and depending on family members’ food preferences. When opting to eat out or have a takeaway, traditional Yemeni food is chosen, and *Adam* and *Sausan* narrate going to Yemeni restaurants, with family and friends, during their non-routine days.

### Home: “Back Home Is Just a Phrase”

During the interviews, British Yemeni young people spoke of home as being a place to relax and at times connect with others through conversing with family. Mealtimes are a key time to do this. *Sausan* looks forward to coming home after a long day at work “to relax”, and during dinner “everyone is home together, eat[ing] together”. *Rashad* returns home from college to “rest” or take a “power nap”, to rejuvenate so that he can go out again in the evening. At the end of one of his more active non-routine days, having climbed Snowdon for charity, *Rashad* “couldn’t wait to get home and just get warm and go to sleep”. Similarly, *Adam* refers to home as being a place to “chill out” but also describes his mother’s cooking, whether Yemeni (or other types of) food as “home food”, connecting home with food and family.

Other references to home, in the context of ancestral homeland (Yemen), were also made during the narratives, particularly when discussions turned to travel, language, and interactions with extended family. The participants had all visited Yemen for holidays.

*Younus: The best thing… was the freedom… even like running around in the neighbourhood, kids were free. Here, it’s like adults need to be on watch all the time in case there is kidnapping or fast cars around. What is nice about Yemen is the nature, and I am an outdoor person, it makes me feel relaxed. It seems fresh and healthy to be in.*

*Rashad: It’s really nice, but it’s a holiday, I guess. I like the weather, the people, the place, the food, the customs. I like going. But when I am there, I do feel different from the Yemenis, the way they act is a little different from me, the things they do… it’s been long since we went, like with the war and all.*

*Fatima: It’s nice and people are friendly and all, but it is just a holiday and then we come back home, and we are back to our normal routines… I don’t feel I am similar to my cousins in Yemen for example. We don’t really know what to talk about when we visit them.*

*Sausan: I love going there during the summer holiday… but it was always just a holiday, and we are outsiders, coming to visit, so I guess it wouldn’t be the same if we lived there long.*

*Nuha: I do feel like that this is my home, I was raised here. ‘Back home’ is just a phrase. Birmingham is my home. It’s what I know*.

Discussion around visiting their ancestral homeland is filled with positivity, but it seems that Yemen is not considered home. When taking a walk-in nature during a routine day, *Younus* refers to Yemen as being a place that is synchronised with nature. He relates his feelings of being “free” in Yemen, a sentiment that may be related to how young he was when he visited Yemen, perhaps more than the actual safety of the country. Unfortunately, due to tribal disputes, continuous civil wars, and poor quality of life (Al-Dawsari 2012; Kaptan 2021), Yemenis in Britain are no longer able to visit their ancestral homeland without war-associated risk.

*Rashad* is the only participant to mention the war in Yemen (Hill 2010) but states that he does not “know much about it”. He also mentioned that his “nan watches it on the news, cos she’s from there”, but that he is “not into all that politics”. Although he acknowledges that the war means that his family can no longer visits, he is not interested in the political state of the country. *Rashad’s* dissociation with matters that are happening in Yemen gives an indication that recent generations have become less affiliated with their ancestral homeland and so are disconnected from the political state of the country.

*Nuha* rationalises this dissociation from Yemen by stating that Britain is “what [she] knows”. *Sausan* adds to this by describing herself as an “outsider” when she visits Yemen, *Rashad* states he is “different” from Yemenis in Yemen, and *Fatima* states that after all, the visits were merely “a holiday”, and after it, they “come back home” to the UK. These conversations suggest that the participants perceive themselves as being more “from here” and that Birmingham is their home. Although they may connect to Yemen in some way or another and enjoy “holiday[s]” in their ancestral homeland, they “come back” to what they know and are familiar with. Furthermore, when *Adam* talks about his future aspirations and wanting to work abroad (to earn tax-free money), he then states that he will then “come back home”. When I asked what he meant by home, he replied: “Birmingham. It’s what you know, what you’re linked to and where you’ve been in”.

### Language: “Arabic Is Part of My History and Culture, and I Like It”

Language is a shared cultural tool reflecting the sociocultural context and connects the individual to society (Hoge 2012). Within the context of this research, the Arabic language also connects the young people to their ancestral homeland as well as other Arab communities that share the same language.

*Sausan: I went to an Arabic school in the weekends when I was young, but I learnt it mainly when I went to Yemen on holidays from time to time. At home we spoke it and read it as well, although most of the time we speak English with each other, even with my Arab friends.*

*Younus: I can read… in Arabic… practice for my second language, so I don’t lose it. My mum, she speaks English, but she would rather speak in Arabic to us, so we, and she, don’t lose the language… when we go Yemen, we can speak to my parents’ grandparents and other family in Arabic.*

All six participants in this study are bilingual and have at some point in their childhood (up until early teens) attended Arabic supplementary schools. This was out of school hours and an educational opportunity for the young people to advance and practise Arabic, hence preserving the language through the generations. *Fatima*, *Nuha*, *Younus*, and *Sausan* narrate how attending supplementary schools was also a way of making friends and to link with those of similar ethnic backgrounds to themselves. *Rashad* states that “most of [his] friends are Yemeni, and come to football, around college, from Arabic school. It’s a nice community thing”. *Sausan* continues to practise Arabic at home and is now working part-time as a tutor of Arabic to younger children. She is proud of being bilingual as it is part of her “history and culture”. However, she finds it easier to speak in English, even with her Arab friends. *Younus* speaks to his mother in Arabic at home, and although he appreciates her efforts and understands the importance of language preservation, he refers to Arabic as a “second language”, suggesting that he is perhaps more proficient in English, having lived in Birmingham all his life. He is interested in learning other languages but tries to keep Arabic a priority so that he does “not lose it”. He is also keen on ensuring that he exercises his brain and suggest that one such way is through practising Arabic. *Younus*, *Sausan*, and *Fatima* also share that, and because they know how to speak Arabic, they can converse with their extended family in Yemen, on the phone or through social platforms. *Sausan* also states that she was able to practise speaking Arabic, when she visited Yemen. This allowed the participants to connect to their extended family through a shared language.

There are also cultural and religious structures connected to Arabic, and the interaction between language and religion as a sociolinguistic field of study is ongoing (Alsaawi 2020). Arabic is the language of the Quran, and so learning it becomes part of the religion (Ediyani 2020). *Fatima*, *Nuha*, *Sausan*, and *Younus* reported that their daily activities involved reading the Quran, a habit they had developed over the years, and that they were able to do so because they could read Arabic. The young people also used Arabic terms that were affiliated with Islamic traditions during their narrative. Examples include “Inshallah” when talking about actions intended in the future, “SubhanAllah” when amazed by something, “Wallah” when swearing in the name of God, and “Alhamdulilah” when pleased about something or feeling blessed. These Arabic terms also reflect the Islamic concept that actions are done by the will of Allah (Uddin and Mazumder 2014).

During conversations, the participants also switched between English and Arabic, some more evidently than others. I understood these terms and sentences, being bilingual myself, and was able to translate the Arabic references to English. There are challenges to translating qualitative data from Arabic to English (Almahasees 2017) and in the absence of a standard way for translating cross-linguistic qualitative research, I used direction from Kashgary (2011) and Mosca and Bot (2017) when translating, paying close attention to certain points in the text as well as enhancing cross-cultural awareness. The terms used by the participants were also in a Yemeni dialect, rather than traditional Arabic, and so to check I had understood the intended meaning, I asked other Yemeni adults, at a local community centre, to translate the terms.

*Rashad*, *Younus*, and *Adam* used more explicit Arabic words to describe concepts and feelings more readily. Such words, found in bold below, are not only terms and texts in Arabic, but also carry an expression of emotions.

*Rashad: I put my head down, three months and said like khalas, and just did it*.

*Younus: Like, they don’t want me to do things against their culture. Cos it would be like Ayb. We don’t do many things that are rude, and so we don’t usually use the word Ayb, but it is often like to things that go against their culture. Like, food etiquette and the way you sit down, should be a respectable manner, like spreading legs in front of my mum or dad. Simple things, that don’t usually bother me so why not keep to them to please my parents.*

*Adam: I am in charge of my own actions, if I like died today, we are going to God, and I can’t say ana disht samihnee. Enough. I’m not like a violent person, but I used to see myself as I’m a nice guy, but ana mush ahbal.*

*Rashad* spends most of his days planning his meals or exercising to keep fit. He declares that the decision to lose weight emerged from the negative feelings he had of himself, of how he looked being overweight. The word “khalas” has different meanings but must be understood through the context and situation. In this situation, *Rashad* uses the word to mean “that’s enough” to suggest that he is tired of his overwhelming situation of being overweight, and the term here is more accessible, to *Rashad*, in Arabic than in English. Similarly, *Younus* uses the term “Ayb” to describe some activities that are (to Yemenis) culturally insensitive, and although roughly denotes shame, disgrace, and dishonour (Al Jallad 2010), the term here implies activities that go against *Younus’* family culture. *Younus* associates these activities to his parents’ culture, rather than his own practices and culture.

*Adam* narrates his experience of being a part of a gang. He uses a combination of Arabic words “but ana mush ahbal” (which roughly translates as “I am not stupid”) to describe how he alerted himself to his situation, describing it with strong emotions. He speaks about religious and moral reasons as to why he is no longer in a gang, “ana disht samihnee” (roughly translates as “I am reckless/imprudent, forgive me”), and expresses his feelings about this situation in Arabic more readily. This combination of words can only be understood in the context of the conversation and is far more understandable and meaningful, to *Adam*, in Arabic. As Arabic and English are of different and distant origins, the meanings that *Adam* wants to convey may be lost in translation if said in English. Therefore, the Arabic texts used may connect to various social and cultural elements of his lived life that provide a contrast, a distinction from his former aggressive self being a member of a gang.

### Religion: “Islam Makes Sense, and It Is a Lifestyle”

All participants in this study identified themselves as Muslims and carried out activities, such as prayer or reading the Holy Quran. *Fatima*, for example, reads the Quran because she is “accustomed to it. Like it’s just a routine thing that [she does]” and expresses a form of discomfort if she does not do so: “I would feel like a gap somewhere in my day and that would be unsettling”. She also states that she has prayed in “changing rooms when shopping”. Other participants do not relate routine religious practices as such, but they do mention some form of connection to God, either through using terms such as Inshallah (God willing), or as in *Adam’s* case, using Arabic to rebuke himself having shame in the face of God, for his previous gang-member-associated activities, as discussed earlier.

The narratives also connect religion with other themes. For example, the significance of Friday to the Muslims community is reported in *Nuha*, *Rashad*, and *Adam*’s narratives. On Friday, if members of the community are not in school or working, they observe a communal prayer Friday prayer, known as Jumma. They may also gather for dinner with their family, enjoy Yemeni cuisines and socialise with one another. Friday is also part of the weekend in Yemen, and so social cultural practices around this day may have originated from family history in the ancestral homeland yet are also connected to the religious significance of Friday.

*Nuha: The bread smells lovely… every Friday usually we all sit together. Like a tradition… that day we had kabsa and aseed… we eat every Friday together as a family. We also do bakhoor, especially on Friday.*

*Fatima: we see my mum’s side once a week when we have a family gathering on Fridays which is nice and very chilled out. We eat together on that day and chat and play cards, like that... we usually do a pray too.*

*Rashad: This is a Friday and we had it off during the college holidays. It’s a Jumma day. So, I wake up... I take a shower and get ready for Jumma… And Jumma is like a communal prayer so go and see others. So, I went to Jumma… its only like half an hour, so I might meet some of the regular friends that I play with football.*

*Nuha* focuses on the cultural significance of Fridays in getting together as a family to eat traditional food, such as “kabsa and aseed” and perfume the house with “bakhoor”, and this, she states, is “like a tradition”. *Fatima* echoes the feeling of a social family get together that she experiences when she visits her mother’s side of the family. Friday also coincides with the English start of the weekend which makes it easier for such meetings. *Rashad*’s experiences of Friday are also linked to socialising and meeting friends, even if it is in the Islamic context of taking part in the Friday prayer. The community youth club which he attends also runs on Friday evening.

There is also a sense of purpose linked to performing religious activities, and the participants were keen on displaying Islam positively, differentiating between it and some misconceptions and misrepresentations which they felt were presented in the media.

*Adam: I feel like Islam doesn’t tell you just to pray five times a day and just orders, Islam is like a way of life, it even tells you how to wipe your ass and with what hand, you know the detail. It tells you through the Prophet’s teachings Hadith and Prophet teachings, its internal as well so your life is happier. It’s complete*

*Younus: Islam is not like just a religion, it makes sense, and it is a lifestyle… there is misrepresentation of Islam..., it’s all ignorance. Those that are not Muslim, don’t know what to believe, and those that are, some, just represent it wrongly. And only if you want to understand the religion, you need to read it for yourself and be your own judge… the laws of Islam, and it makes sense, otherwise I wouldn’t do it.*

*Nuha: Why do you need to sleep around first to check who is the right guy? I don’t mind the Islamic part, because it makes sense in the long run, and the boundaries are set that make sense… I do believe I am a religious person, more than culturally. I try not to let my culture hold me back, and embrace its positivity and uniqueness, but my religion is my direction I feel.*

*Younus*’s activities are driven by his religion and as he feels they “make sense”. *Nuha* agrees with the “Islamic part” of the rules in Islam and justifies her actions and being in relation to her faith. *Younus* feels that there is a need to differentiate religion from aspects of his Yemeni heritage and culture or social expectations within the community, and religion seems to be basis of his beliefs and activities. Anything which is not part of religion is diminished. For example, when he talks about growing his hair long, his father disapproves, and *Younus* states that this is “a cultural thing” as the father “thinks it’s more for girls”. *Younus* feels that, so long as it is not against his religion, he will continue having long hair. This shows some independence of thought and demonstrates how he prioritises his religious beliefs over his family’s culture and ideologies. *Younus* does not agree with, or follow some of his parents’ opinions, and states: “They have their way and I have mine”. This shows some level of disconnection that *Younus* feels towards some elements of his own family history and practices.

## Discussion

The thematic analysis of the sample of British Yemeni young people highlighted certain common aspects of Yemeni subculture that appear embedded in their conduct of everyday life. And yet for each person, such themes have a different nuance, in particular how they are either different to and/or become assimilated into their understanding of mainstream British culture. In the “Discussion” section, I focus on this cultural hybridity of living as a key element of understanding the identity, practice, and personhood/personality of British Yemeni young people—one that enables a theoretical examination of such a combination.

Through the lens of lived experiences, the cultures and subcultures of British Yemeni young people were observed. I understand mainstream culture to convey the culture in which the young people live within and practice, that is the British culture that they were born into and associate many of their social practices with. Such mainstream culture reflects a shared meaning-making that is understood and interconnects their lived lives with societal norms and values. Additionally, the young people also have a Yemeni subculture that they, at times, are associated with and to, notable through the five themes discussed here. This subculture also connects to localised community and shared values, norms, and orientations in those communities. It is often represented through family histories and practices. Culture links people with community, both British and Yemeni, and the community with the nation (Shah 2016), and so in understanding the conduct of everyday life of British Yemeni young people, it was possible to articulate their relational cultural psychological experiences.

The conduct of everyday lives highlights the hybridity and perhaps aspects of translations or adaptation from and to both mainstream British culture and Yemeni subculture. This paper has shown how the young people’s social practices have historical dimensions that are fluid and can create an insight into the different personalities and identities of Yemeni young people in Britain through engaging with both British mainstream cultures and Yemeni subcultures. The similarity or dissimilarity between the culture of settlement and the culture of ancestral homeland influences the personality structure of the young person as well the construction of cultural identity (Bhui et al. 2005). For example, *Rashad* attends communal Friday prayers when he is not at college, and this links to his religious social identity structure as well as his need to establish an ethnic cultural identity through communicating with people of, perhaps, similar ethnicity and religious identity. Rashad states that during the Friday congregation, he “meets some of the regular friends that [he] plays football with. They are all Yemenis”. This to *Rashad* is perhaps a way of constructing elements of his cultural identity.

Where Yemeni practices meets British practices is evident, and parts of the narratives show a combination of both cultures. For example, Younus enjoys many different cuisines, “including traditional fish and chips” as well as Yemeni cooked food. *Adam* is particularly fond of Yemeni meals at the weekend, which he enjoys with his friends at a local Yemeni restaurant. Traditional Yemeni cuisines are accessible in Birmingham, and this is perhaps a way in which the Yemen community preserves traditional culture and heritage while in Britain. *Adam* and *Sausan* dine at Yemeni restaurants, and the presence of such restaurants represents a form of regional identity, suggesting the presence and active environment of the current Yemenis in this region of the UK. It may also be a mode of adaptation for Yemenis in Britain.

There is other evidence in the young people’s narratives that their practices reflect the construction of a cultural hybridity, providing a space for personal and collective experiences of cultural values and national interests (Burke 2012). The shared food choices, the attempt to preserve the Arabic language, the focus on physical image and acceptance in society, and the practices associated with religious connections together show an integrated evolving culture of the participants being influenced by both British mainstream culture and Yemeni subcultures. Cultural hybridity thus communicates an amalgam of themes, influenced by both heritage and practices related to their ancestral homeland, as well the society in which they live. Both cultures are held in important and are more apparent in various everyday transactions in particular societal settings.

Furthermore, the similarities across the six participants suggest that the Yemeni communities in Britain share common historical experiences, cultural beliefs, and family practices, and they have a broad collective consciousness of belonging together. For the young people, these collective beliefs and practices are also affected and influenced by living in and within the British society. Within the five themes explored in this paper, there are also differences in the specifics of experiences, but these are interconnected and meaningful at the individual and community level. In terms of preserving certain practices and traditions of the ancestral homeland culture, the six case studies showed efforts to preserve Yemeni subculture, such as cooking Yemeni food, sitting on the floor during dinner, going to Arabic supplementary schools from a young age, visiting Yemen (particularly before the war), practising the Islamic faith, and switching languages in conversation. The participants’ parents often took them to Yemen for holiday and these trips, or “ethnic pilgrimage” (Kelly 2000), may have contributed to the construction of their culture and ethnicity (Nagel 1994).

Home, however, is considered to be Britain, the country they associate most with. Terms such as “back home” are unambiguous in their interpretations. They all connect home to the place they live and when talking about visiting Yemen acknowledge that it is “just a holiday” and “home is Birmingham”. This shows complete disassociation with Yemen being home, and this is the case with all participants, whether first, second, or third generation. Although the young people have a romanticised experience of Yemen—“the weather, the people, the place, the food, the customs” all being “nice” and “fun”—the reality is that Britain is what they know as home.

There is also an overarching sense that bridges these themes, showing hybridity as integrated and interconnected than merely either/or. The activities and meaning associated with the activities of the participants show that they connect and disconnect to both mainstream British culture and the Yemeni subculture. They connect to certain rituals around the main themes explored here but at the same time disconnect from other aspects of subcultural identities. *Sausan*, for example, connects to certain rituals related around body image and what is perceived as beauty through the application of makeup to suit young mainstream British society and structure in which she is part of but disconnects with such culture by mainly following social media that links to her religious affiliation of being a Muslim hijab-wearing woman. *Younus* connects to family time, elements around food that relate to his ethnicity and religion, such as eating with his hands, or on the floor, “the Yemeni way”, but disconnects with such culture at different times by eating fish and chips or sitting at the table to eat, for example. When he visits Yemen as a child, *Rashad* connects to some of the enjoyable experiences of being in Yemen but disconnects from the actual realities of Yemeni life as it is now. Within the narratives, there is a romantic reflection of the cultural heritage of Yemen located in the participants’ culture, connections, and networks, and so it becomes embedded as part of the individual.

At the community level, the Yemenis in Birmingham connect with mainstream British culture by carrying out various day-to-day activities within social contexts (university, colleges, work) and social arrangements to suit the culture in which they live. However, they disconnect with this by associating with the subculture of Yemen by, for example, attending Yemeni restaurants and community centres where Friday congregation prayer are performed, as a manner of adaptation in Britain, and as an expression of minority, religious and ethnic identity (Rijal 2009). The participants’ parents also connect with Yemeni subculture by, previously, taking their children to visit Yemen to ensure that they experience, at a personal level, their ancestral homeland, appreciate its history, language, and culture through a subjective constructive lens and experience life with people in Yemen. However, the young people disconnect with Yemen by not (noticeably) exposing the current war situation happening in Yemen. Only *Rashad* mentions the war in the interview, and only as a side remark, dissociating himself from the matter by stating that he is not “really into politics”. The participants also connect with mainstream British culture by speaking English as their main language, even with Arab-speaking friends, as evident in *Sausan’s* narrative and in *Younus*’s description of Arabic as a “second language”. They find themselves at the core of a complex web of power relations which potentiates their production of multilingual practices (Moraru 2019). The parents also connect with Yemeni subculture by ensuring their children learn Arabic—they take them to supplementary schools, and visiting Yemen before the war, and by speaking to them in Arabic at home. This connection and disconnection occur in different contexts for different reasons and provide an emerging sense of British Yemeni youth culture, evolving and developing as cultural identities associated with first, second, and third generations of Yemeni young people in Britain.

The examples discussed in this paper show that it is in the young people’s evolving conduct of everyday life, the living and being is embedded in social practices around families and communities that have histories, and that they are also part of. This produces the cultural lives and living of not only the young people, but also the communities they are within. It is in the experiencing and doing and living in such contexts that researchers can see certain themes emerging of what is important in that everyday life, both in terms of evolving everyday habits and regular interval-led habits such as holidays and time-outs. The everyday doing of culture is evolved and transformed by the young person, and so the cultural hybridity of British Yemeni young people is not fixed but rather an evolving personhood. Using the conduct of everyday life does not hold up a set of cultural values and norms over young people as separate which then inculcates/socialises them in some sort of way. Rather, it is the connecting networks of experiencing and doing that provides the essence of who these young people are. This may explain, for example, why the war in Yemen is of little importance, unless it directly impacts the conduct of everyday life in particular ways it will remain irrelevant to the young people.

## Conclusion

This paper examines the experiences that are similar across the six British Yemeni young people and notes the five main themes that emerge from the data. Through the study of their activities in the context of the conduct of everyday life, these themes—body image, food, home, language, and religion—provide a sense of how the young people have interacted, participated, moved, and engaged in the order and arrangements of their living and being. Of particular interest in this paper is the extent to which these young people’s family histories and practices influence their conduct of everyday life and what is happening in the lived lives of these individuals and how using the conduct of their everyday living enables the themes to emerge. The way British Yemeni young people connect and disconnect various times and places to mainstream British culture and Yemeni subculture gives an indication of the emergence of their unique hybrid culture.
